# Self-Assembling Enzymatic Nanocomplexes with Polypeptides and Low-Weight Organic Compounds: Preparation, Characterization, and Application of New Antibacterials

**DOI:** 10.3390/ijms24031831

**Published:** 2023-01-17

**Authors:** Ilya Lyagin, Nikolay Stepanov, Denis Presnov, Artem Trifonov, Elena Efremenko

**Affiliations:** 1 Faculty of Chemistry, Lomonosov Moscow State University, 119991 Moscow, Russia; 2 Skobeltsyn Institute of Nuclear Physics, Lomonosov Moscow State University, 119991 Moscow, Russia; 3 Faculty of Physics, Lomonosov Moscow State University, 119991 Moscow, Russia

**Keywords:** nanomaterial, enzyme, molecular modeling, quorum quenching, antibacterials, penicillin acylase, hexahistidine-tagged organophosphorus hydrolase, emodin, polymyxins

## Abstract

The self-assembling of nanosized materials is a promising field for research and development. Multiple approaches are applied to obtain inorganic, organic and composite nanomaterials with different functionality. In the present work, self-assembling nanocomplexes (NCs) were prepared on the basis of enzymes and polypeptides followed by the investigation of the influence of low-molecular weight biologically active compounds on the properties of the NCs. For that, the initially possible formation of catalytically active self-assembling NCs of four hydrolytic enzymes with nine effectors was screened via molecular modeling. It allowed the selection of two enzymes (hexahistidine-tagged organophosphorus hydrolase and penicillin acylase) and two compounds (emodin and naringenin) having biological activity. Further, such NCs based on surface-modified enzymes were characterized by a batch of physical and biochemical methods. At least three NCs containing emodin and enzyme (His_6_-OPH and/or penicillin acylase) have been shown to significantly improve the antibacterial activity of colistin and, to a lesser extent, polymyxin B towards both Gram-positive bacteria (*Bacillus subtilis*) and Gram-negative bacteria (*Escherichia coli*).

## 1. Introduction

Bacteria use various systems of Quorum Sensing (QS) [1] and bacteria of the same genus can preferably utilize similar signal routes. *N*-acyl homoserine lactones and pheromone-like peptides are among the most well-known QS inductors of Gram-negative and Gram-positive bacteria, respectively. In the case of Quorum Quenching (QQ) of Gram-negative bacterial cells, the action of enzymes hydrolyzing the *N*-acyl homoserine lactones appeared very effective, and the combinations of the QQ-enzymes with antibiotics significantly improves the efficiency of the latter [2,3].

Additionally, antimicrobial polypeptides play a dual role in such combinations with QQ-enzymes: they retain their antimicrobial properties and act as stabilizers for enzymes owing to the multiple surface intermolecular interactions [3,4]. The enzymes also usually have dual functions in such combinations with antibiotics: they reduce the resistance of the cells against the action of the antibacterial compounds by QQ and play the role of vehicles for the antimicrobial polypeptides, preventing their hydrolysis by bacterial proteases.

Another approach to reducing QS functioning is based on interfering with it through the application of inhibitors. For example, the QS system *agr* in bacterial strains from the genus *Staphylococcus* (including *S. aureus*, *S. epidermidis*, etc.) is the most known and investigated one to date [5]. Its receptor—AgrC—can be inhibited by natural and synthetic peptides with a linear structure [6] and their derivatives; by cyclodepsipeptides [7] and their derivatives; by peptoids [8], etc. 

Analogously the *fsr*-system in *Enterococcus faecalis* [9], VirSR in *Clostridium perfringens* [10], ComD in strains from the genus *Streptococcus* [11], PlcR in *Bacillus cereus* [12], etc., can be modulated artificially. The wide diversity of QS receptor proteins does not allow *a priory* relying on the same effector to bind to all possible targets. Though, as an exception, rarely universal mimetic inhibitors are possible [13].

**Figure 1:**
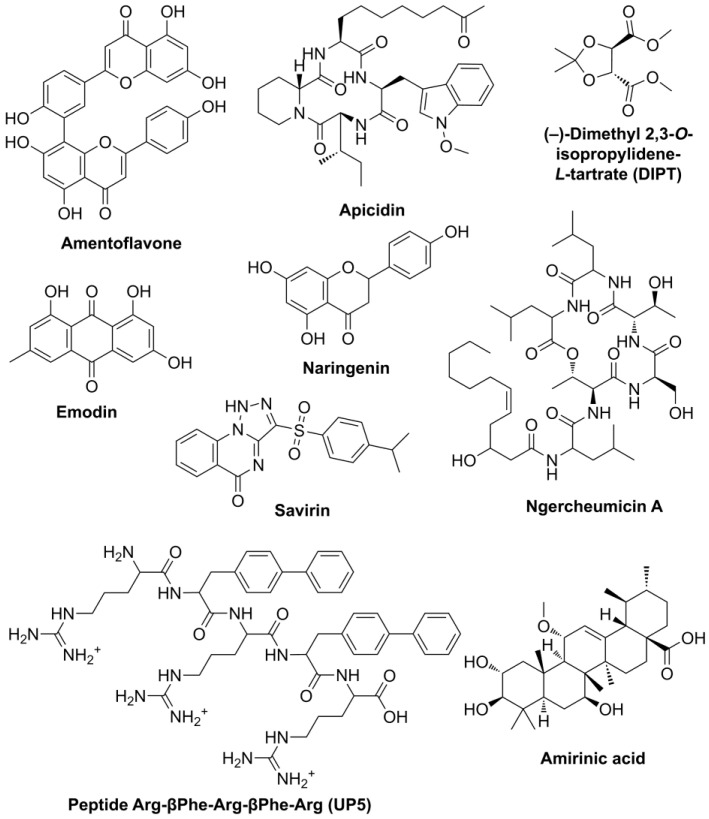
Chemical structures of selected substances interfering with QS systems of Gram-positive bacteria.

Screening and molecular modeling of binding modes of potential QS inhibitors are mainly concentrated on their interaction with target receptors currently [14]. As opposed to that, the main criterion for selection in the current work was the possibility of an unhampered combination of effectors for various targets within single preparation. Namely, several compounds of different chemical structures which are able to interfere with the QS-systems of Gram-positive bacteria were selected from the literature: amentoflavone [15], apicidin [5], (–)-dimethyl 2,3-*O*-isopropylidene-*L*-tartrate (DIPT) [16], emodin [17], naringenin [5], ngercheumicin A [7], savirin [5], peptide Arg-βPhe-Arg-βPhe-Arg (UP5) [18] and amirinic acid [19] (Figure 1).

The binding of polypeptides [20] or low-weight organic compounds [21] to enzyme surfaces can form nano-sized complexes (i.e., nanocomplexes, NCs). Historically the most useful methods to investigate such interactions were optical techniques which can be applied to both low-weight organics [22] and inorganic nanomaterials [23]. Atomic Force Microscopy (AFM) allows observing self-assembled enzyme NCs with polymers, e.g., block-*co*-polymer of polyethylene glycol and polyglutamic acid (PEG-PLE_50_), directly [24] in a dried form or in a solution. Therefore, such analysis could additionally provide straightforward evidence for NCs formation, including QS effectors.

In this work, interactions of QS effectors with a number of QQ enzymes (His_6_-OPH, penicillin acylase, carboxypeptidase A and thermolysin) having hydrolytic activity towards *N*-acyl homoserine lactones (i.e., QS inductors of Gram-negative bacteria) [20] or peptides were modeled. On the basis of simulations, the appropriate ones were rationally selected and their NCs were characterized by AFM and (nano)particle tracking analysis (PTA). Finally, the antibacterial activity of NCs was investigated in Gram-positive (*Bacillus subtilis*) and Gram-negative (*Escherichia coli*) bacterial cells in the presence and absence of effectively acting antimicrobial polypeptides (polymyxin B and colistin). The effectiveness of the antimicrobial action of triple NCs composed of QS effector, QQ enzyme and the antibiotic compound was evaluated for the first time, applying luminescent ATP-metry.

## 2. Results

### 2.1. Molecular Modeling of Interactions of Enzymes with QS Effectors

**Figure 2:**
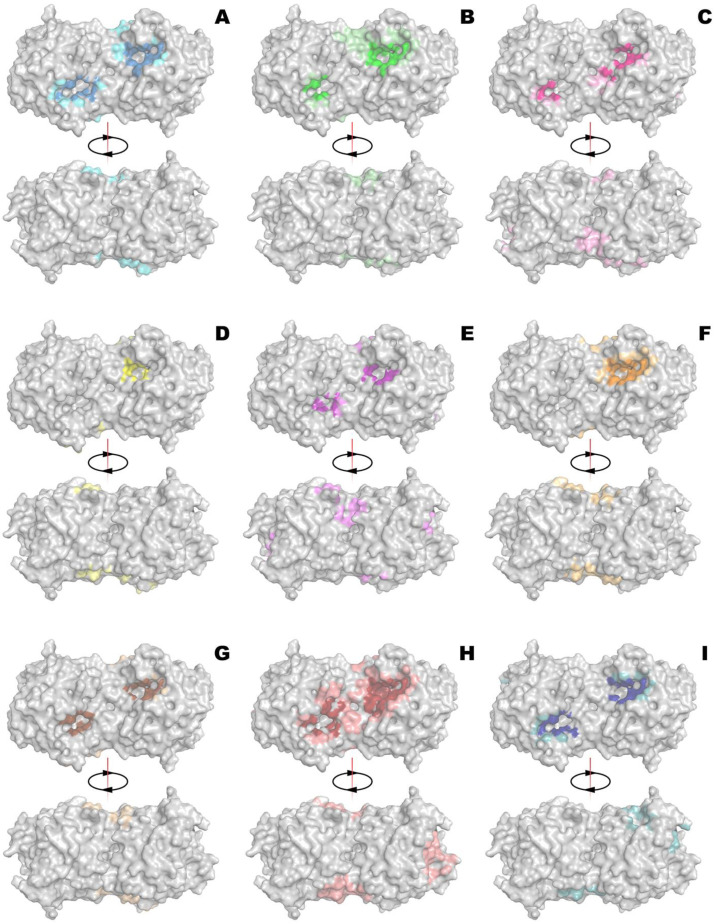
Binding of amentoflavone (**A**), apicidin (**B**), DIPT (**C**), emodin (**D**), naringenin (**E**), ngercheumicin A (**F**), savirin (**G**), UP5 (**H**) and amirinic acid (**I**) to front and back side of His_6_-OPH. Occupied surface near active site is highlighted by more intensive color. The top binding poses of emodin and naringenin near active sites are illustrated on Figure S4.

**Figure 3:**
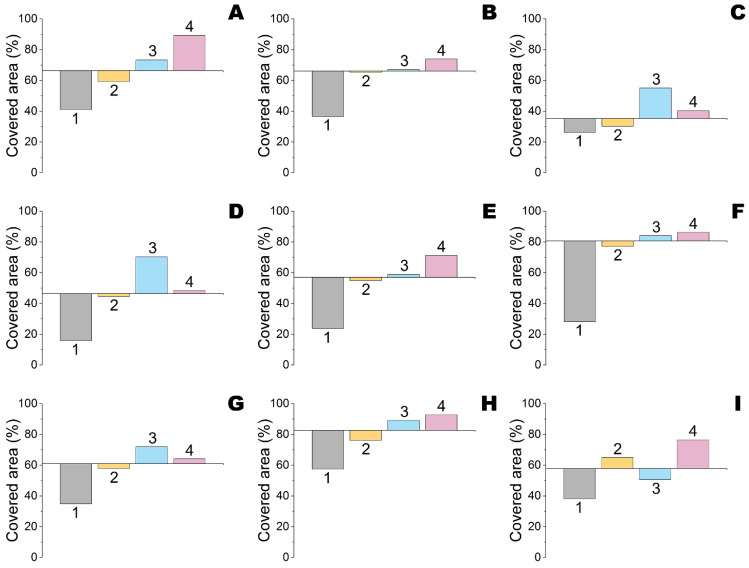
Relative surface near active sites of His_6_-OPH (#1), penicillin acylase (#2), carboxypeptidase A (#3) and thermolysin (#4) occupied by amentoflavone (**A**), apicidin (**B**), DIPT (**C**), emodin (**D**), naringenin (**E**), ngercheumicin A (**F**), savirin (**G**), UP5 (**H**) and amirinic acid (**I**) at pH 7.5. An interception of *x*-axis is equal to a median value for all four enzymes interacting with the certain effector under consideration.

Nine compounds (QS inhibitors) selected for computational analysis had multiple binding modes with the surfaces of four QQ enzymes at pH 7.5 (Figure 2 and Figures S1–S3). The most prominent and important ones were located near the active sites of the enzymes. It meant these QS inhibitors can affect catalytic activity to a varying degree: the wider (and deeper) the occupied surface and the stronger the interaction, the more hindrances for substrate access would arise. Analysis has shown that minimal occupation of active sites by all investigated effectors was in the case of His_6_-OPH (Figure 3, Table S1), followed by penicillin acylase (with an average difference of 24%). At the same time, thermolysin was maximally occupied by most effectors. The maximal coverage of active sites of all enzymes was observed for ngercheumicin A and UP5 (up to 93%). Interestingly, the binding of His_6_-OPH with UP5 was even larger (near the active site and totally), than with amentoflavone which is known to decrease enzyme activity on ca. 20% at pH 7.5 [21]. The minimal coverage of active sites of all enzymes was determined for DIPT and emodin. The last one was the best choice to combine with His_6_-OPH and followed by naringenin. From a binding energy standpoint, the strongest interaction (ca. −8.8 kcal/mol) was in the case of amentoflavone with His_6_-OPH, penicillin acylase and thermolysin (Table 1 and Table S2).

The weakest binding with all enzymes was revealed for DIPT (ca. −4.2 kcal/mol). Furthermore, all four enzymes were undistinguishable in this case. That was quite strange since DIPT occupied variable areas on the surfaces of these enzymes and should be investigated with a wider number of QQ enzymes in future work(s).

As a result, two QQ enzymes (His_6_-OPH and penicillin acylase) and two QS effectors (emodin and naringenin) were selected for further experiments in vitro.

### 2.2. Influence of Selected QS Effectors on Self-Assembling of Enzyme NCs

**Figure 4:**
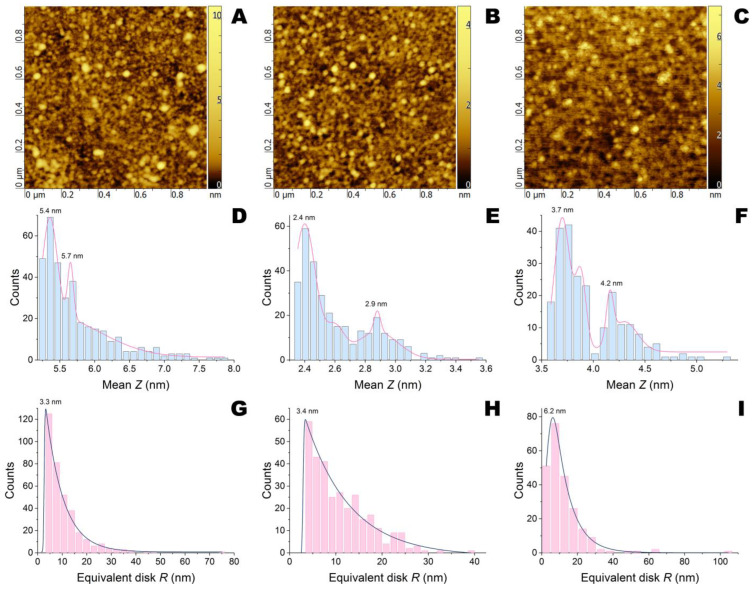
AFM images and their statistical treatment for APS-modified silicon wafers after application of penicillin acylase/PLE_50_ without QS effectors (**A**,**D**,**G**) and with emodin (**B**,**E**,**H**) or naringenin (**C**,**F**,**I**).

**Figure 5:**
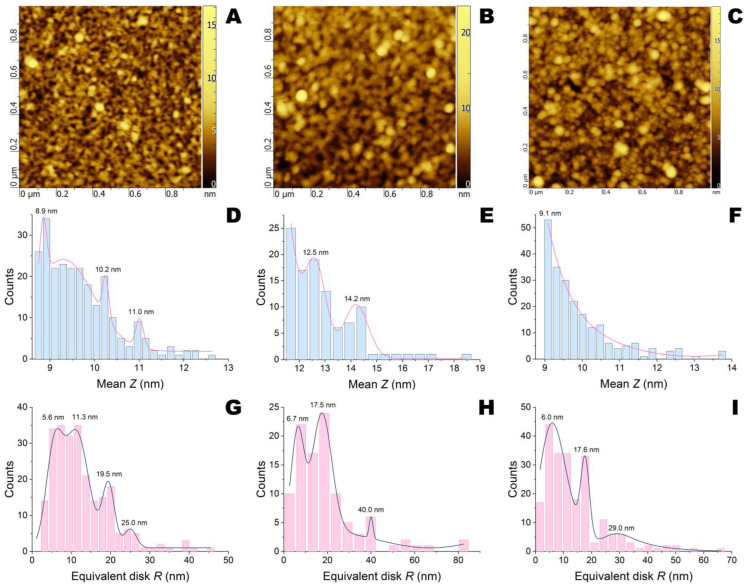
AFM images and their statistical treatment for APS-modified silicon wafers after application of His_6_-OPH/PLE_50_ without QS effectors (**A**,**D**,**G**) and with emodin (**B**,**E**,**H**) or naringenin (**C**,**F**,**I**).

To visualize the NCs of enzymes with QS effectors, initially, such complexes with polypeptides were obtained and then the structure of self-assembled NCs with or without QS effectors was investigated by AFM (Figure 4, Figure 5 and Figures S5–S7). According to the work [25], modification of silicon wafers by 3-aminopropyltrimethoxysilane (APTMS) under selected conditions gives a monolayer 3-aminopropylsilane (APS) structure which was confirmed by a smooth surface with narrow grain distribution (Figure S5). Application of negatively charged NCs with PEG-PLE_50_ which were investigated similarly [24], resulted in a dramatic increase in both the height and radii/diameters of grains (Figure S6). Additionally, there was an excess of grains of 40–60 nm diameters and 3.9–5.5 nm heights. These values were comparable with previously established characteristics of 29–44 nm diameters and 4–7 nm heights for NCs of His_6_-OPH/PEG-PLE_50_ [24].

The dimensions of NCs with succinylated gelatin (gelofusine), which was used as a negative control since it significantly blocks the active sites of both penicillin acylase and His_6_-OPH [26,27], depended on the enzyme applied (Figure S7). Thus, the NCs of penicillin acylase/gelofusine were wider (40–100 nm) but shorter (5.2–6.6 nm) compared to His_6_-OPH/gelofusine (40–70 nm diameters and 5.9–8.1 nm heights). The NCs of penicillin acylase/PLE_50_ had comparable dimensions (40–80 nm diameters and 5.2–7.5 nm heights) compared to penicillin acylase/gelofusine (Figure 4). At the same time, the NCs of His_6_-OPH/PLE_50_ (40–80 nm diameters and 8.6–12.2 nm heights, several populations) were found to be much taller than His_6_-OPH/gelofusine (Figure 5).

The addition of QS effectors had a different influence depending on the chemical structure of the compound and enzyme NCs. Both emodin and naringenin decreased the height of penicillin acylase/PLE_50_ grains to 2.3–3.2 and 3.6–5 nm, respectively, but had little or no effect on diameters. Moreover, the splitting into two height populations was profound in both cases.

The addition of emodin to His_6_-OPH/PLE_50_ increased both diameters (up to 160 nm) and heights (up to 15 nm) while preserving the population pattern. Naringenin had no effect on the sizes of His_6_-OPH/PLE_50_ but smoothed its height distribution.

Hydrodynamic sizes of various NCs without QS effectors were undistinguishable for both penicillin acylase and His_6_-OPH (Table 2, Figures S8 and S9). 

Very little (or even no) differences were determined for minor fractions also. A dramatic increase in nanoparticle sizes and changes in population structure was observed during the addition of emodin and, to a lesser extent, naringenin to penicillin acylase/PLE_50_ (Table 2, Figure S9). Such structural shifts were lower in the case of His_6_-OPH/PLE_50_ but still detectable. Thus, the fraction of large-size His_6_-OPH/PLE_50_ nanoparticles (ca. 160 nm) increased steadily (Figure S9).

Interestingly, emodin having intrinsic fluorescence significantly increased the sensitivity of the detection of enzyme NCs. Moreover, part of the nanoparticles became fluorescent which meant the binding of the QS effector to them. 

Though there was no such purpose in the work and modification(s) in the current device configuration will be necessary to enumerate such nanoparticles quantitatively, it could be exciting to study such NCs with different fluorescent compounds in the future.

### 2.3. Influence of NCs Assembly with Selected QS Effectors on Enzyme Activity

**Figure 6:**
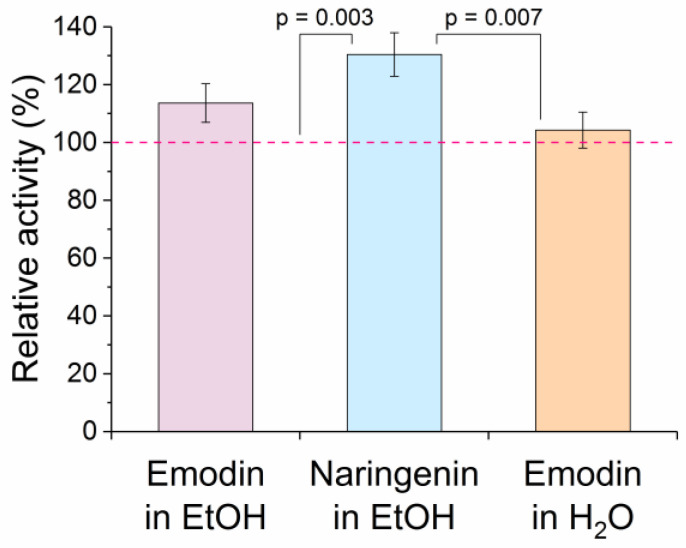
Influence of ethanol and water solutions of emodin as well as ethanol solution of naringenin on activity of His_6_-OPH. Activity of the enzyme under the same conditions (i.e., with 0.5% EtOH but without effectors for alcohol solutions) was considered 100% (dashed line). Emodin and naringenin were added into reaction mixtures at a ratio of 2 and 18 molecules per His_6_-OPH dimer, respectively. There was a statistically significant difference (N = 3, one-way ANOVA, *p* = 0.002) and pairs within the significance level (0.05) are labeled. Differences for all other pairs were statistically non-significant (*p* > 0.05) by the Holm–Sidak method of multiple pairwise comparisons.

The influence of ethanol solutions of emodin and naringenin on the enzyme activity of naïve His_6_-OPH was investigated (Figure 6). It was shown that the activity of the enzyme did not decrease and even more His_6_-OPH was stabilized against the inactivating action of ethanol. That resulted in an apparent increase (up to 30%) compared with controls lacking emodin or naringenin. 

### 2.4. Antimicrobial Activity of Selected NCs

Initially, the antibacterial activity of emodin and naringenin alone was investigated in Gram-positive (*Bacillus subtilis* B-522) and Gram-negative (*Escherichia coli* DH5α) bacterial cells (Figure S10). As expected, both QS effectors did not have prominent antibacterial activity.

**Figure 7:**
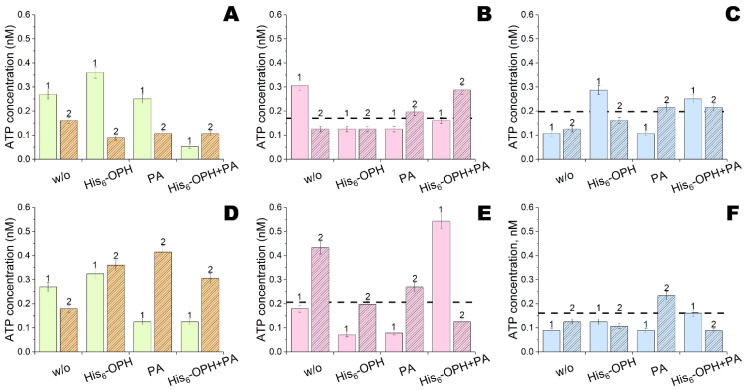
Antibacterial activity of emodin (#1) and naringenin (#2) NCs with His_6_-OPH and/or penicillin acylase (PA) towards *B.subtilis* (**A**–**C**) and *E.coli* (**D**–**F**) cells without any additional antibiotics (**A**,**D**) and with addition of colistin (**B**,**E**) or polymyxin B (**C**,**F**). The levels of ATP with these antibiotics alone are labeled with dashed lines. Emodin and naringenin were applied at a dose of 1 and 10 ng per sample, respectively.

Rather suddenly, such antibacterial activity was revealed for the NCs of emodin and naringenin with both His_6_-OPH and/or penicillin acylase (Figure 7A,D). The maximal antibacterial activity towards both bacterial strains was, in the case of emodin, combined concurrently with both enzymes. It was even higher than the activity of both used antibiotics (colistin and polymyxin B), without any additives and applied at a dose of 100 μg/cm^2^. For comparison, the same efficiency was achieved for NCs with 12 μg/cm^2^ of penicillin acylase and 0.2 μg/cm^2^ of His_6_-OPH. A combination of colistin or polymyxin B with emodin or naringenin in a single preparation improved their antibacterial activity in most cases (Figure 7B,C,E,F).

To potentiate the antibacterial activity of double combinations even more was sometimes possible via the addition of enzymes. However, the contribution of enzymes was rather negligible in most cases. Nevertheless, the maximal efficiency for bacterial removal was determined with NCs of colistin, emodin and His_6_-OPH. This level was achieved with NCs of colistin (or polymyxin B), emodin and penicillin acylase. All these NCs were equally effective towards both Gram-positive and Gram-negative bacteria and could be considered universal.

**Figure 8:**
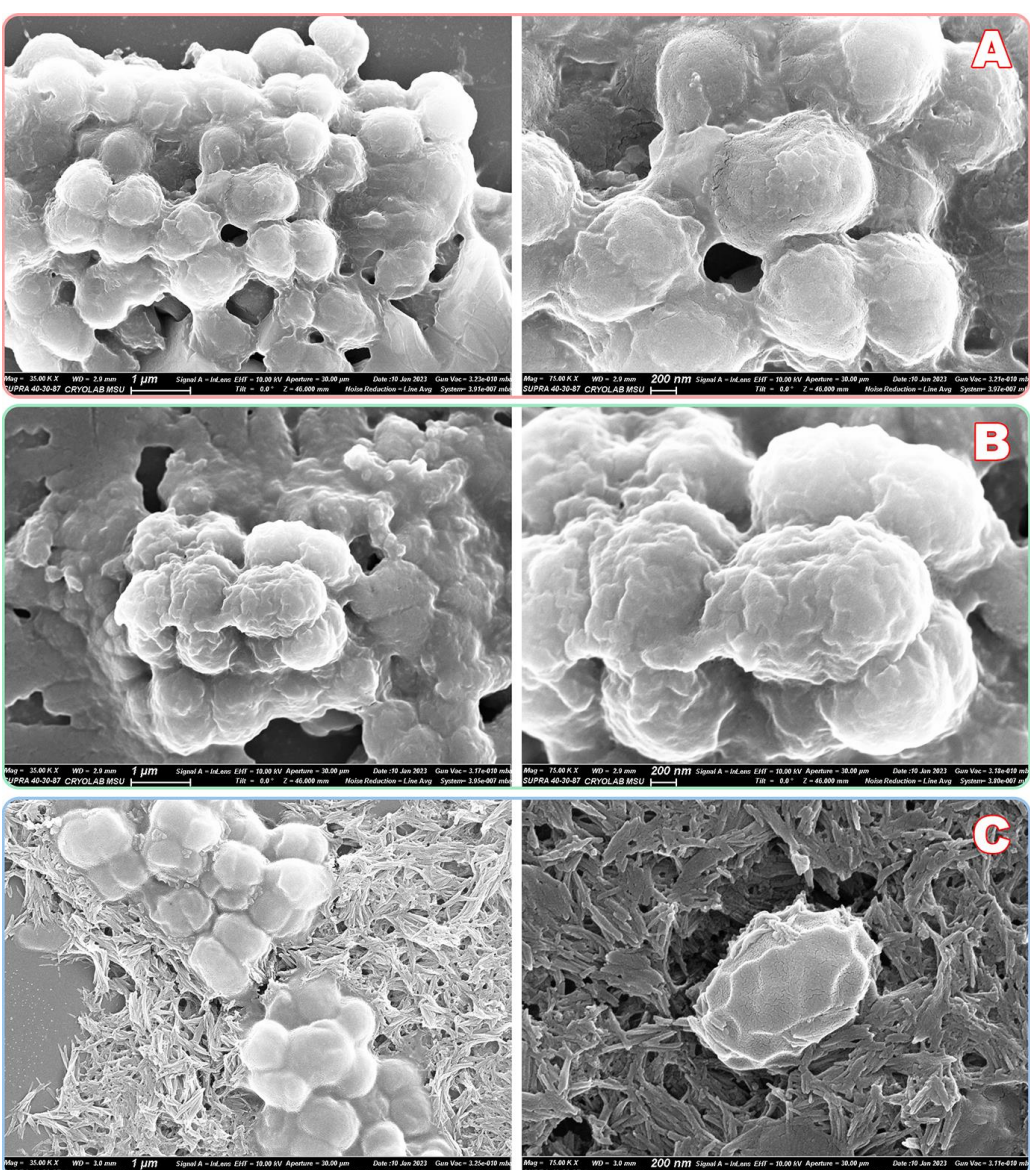
SEM analysis of *E.coli* cells after exposure at 25 °C for 3 h in PBS buffer: control (**A**); NCs of emodin with His_6_-OPH and penicillin acylase (**B**); the same NCs together with polymyxin B (**C**). Cells were treated in suspension and then applied to APS-modified silicon wafers, dried and sputtered by gold.

To elucidate the possible mechanism(s) of the antibacterial action of NCs, *E.coli* cells were also treated in suspension and then visualized with scanning electron microscopy (SEM) (Figure 8). A mature cell culture was present exclusively in the aggregates which were almost uniformly distributed on the surface of the APS-modified silicon wafer and attracted by surface irregularities (e.g., growing crystals of inorganic salts). Both treatments by NCs led to the dramatic extinction of such aggregates. Very few aggregates which were visible by SEM had a lower number of participating bacterial cells. 

Cells were not fixed during the procedure (between exposure and drying step) and thus now it is not possible to conclude whether cell envelopes were deformed ante or post-preparation. However, membrane permeability seems to be increased with NCs and especially with their combination with polymyxin B (Figure 8C).

## 3. Discussion

Communication between cells appears to be a fundamental mechanism for all living beings [28]. During co-evolution, various rival cells could interfere with it while disrupting its steadiness, giving them competitive advantages [29]. However, any resistance to such disruptions and quenching was not formed for billions of years. That should be a solid basis to implement such approaches by humankind in our everyday life. Detailed investigations of different QS systems have already revealed multiple vulnerable targets available for modification and/or inhibition [1]. Thus, mimicking nature’s approach, it is reasonable to combine several activities in a single preparation.

Retention of the catalytic activity of enzymes was the main purpose during molecular modeling. It was possible due to the rejection of unsuitable combinations with highly probable inhibition of enzymes by selected compounds. Particularly, the binding of a QS effector within active site(s) would result in the impossibility of the normal substrate entering the cavity. Most of the screened compounds actually were bound to the active site domains specifically (Figure 2, Figure 3 and Figures S1–S4). In this context, savirin (3-(4-propan-2-ylphenyl)sulfonyl-1H-triazolo[1,5-a]quinazolin-5-one) should be mentioned since it possibly could bind to multiple targets not limited by QS receptors and such behavior could be the reason for its biological activity.

Both proteases were excluded in the first stage as inappropriate for combinations with selected compounds. Moreover, previously, these enzymes have shown rather ambiguous results in combinations with antimicrobial peptides [20]. The other two remaining enzymes are known to hydrolyze *N*-acyl homoserine lactones used by Gram-negative bacteria (but not by *E. coli*). However, they have some modulating effects in NCs with antimicrobial peptides on Gram-positive bacteria also via unknown mechanisms [20].

Confirming the results of molecular modeling, selected compounds—emodin and naringenin—did not decrease the enzyme activity of His_6_-OPH. Furthermore, the activity of His_6_-OPH was apparently increased due to the stabilization of the enzyme in the organic solvent (EtOH) used for the preparation of emodin and naringenin solutions. Similar effects were previously observed for a number of antioxidants with His_6_-OPH and Pr^i^OH or DMSO used as solvents for compounds with antioxidant activity [21].

Naringenin is structurally close to apigenin while differing by only double bond within heterocycle and having comparable stabilization effect on enzyme activity. Structurally relevant compounds for emodin were not issued then. The most profound effect of antioxidants was an increase in the uncompetitive constant of enzyme inhibition by alcohol [21]. It means that emodin and naringenin while binding to His_6_-OPH-like antioxidants can prevent further interaction of alcohol with enzyme molecules at the same site.

There were around 5 and 10 sites on His_6_-OPH and penicillin acylase surface, respectively, to preferably accommodate emodin. The same parameter for naringenin was equal to 7 on both enzymes. Therefore, if these enzymes act as vehicles for QS effectors, they can stably transport, at least, 5–10 mol./mol. (i.e., ca. 26 ± 6 mg/g) into the cell and increase its bioavailability. This can be important since both compounds possess antioxidative properties [30,31] and can be modified off-target. Even more, both compounds can have their own toxicity, e.g., for treated animals and humans. Therefore, more effective transport of QS effectors within enzyme NCs into the target organism(s) would allow for decreasing therapeutic doses.

Triple NCs with polypeptides swelled in solutions with both emodin and naringenin. A twofold increase in mean diameters translated into an eight-fold increase in the volumes of spherical particles. Both QS effectors had binding modes in distal (as compared to the active site) parts of enzyme molecules which are similar to the binding sites with PLE_50_ [26,27]. Competition for the same binding sites would result in the partial detachment of the polymer chain from the enzyme surface, followed by its interaction with another enzyme molecule. Interestingly, larger aggregates have been present, at least, partially within initial NCs of PLE_50_. Moreover, they were retained with emodin and His_6_-OPH/PLE_50_ after drying before AFM. Certainly, such triple NCs are too large from a practical standpoint of easy transportation into bacteria cells and are of scientific interest.

Usually, the efflux pump(s) used by bacteria to remove harmful compounds can eliminate multiple substances and, at least, naringenin [32] thus decreasing its effective concentration. Many studies have successfully employed such competitive interaction to modulate the efficiency of common antibiotics pumped out by the same transporter. Polymyxins used in the current work are generally considered to affect the permeability of bacterial membranes [33] and thus do not require penetration into the cell for their biological activity. However, both emodin and naringenin have shown synergetic effects with polymyxin B towards both Gram-positive and Gram-negative strains and not with colistin at all. These antimicrobial peptides differ by a single amino acid within the macrocycle (Phe in polymyxin B is substituted by Ile in colistin) and by acyl-radical (longer by single carbon in polymyxin B). Further modification(s) of the macrocycle and qualitative structure–activity investigations could reveal possible pathways to improve the antibacterial activity of such combinations.

Interestingly, preferences were slightly shifted to emodin and colistin when combined with any enzymes (Figure 7). It seems the binding of naringenin to penicillin acylase reduced the (bio)availability of the QS effector for stimulating the activity of the antimicrobial peptide. Meanwhile, such an effect was lesser with His_6_-OPH.

Currently, most R&D with nanocomposites is dominantly focused on medicine [34], including combinations with enzymes [35]. The same approach is successful when it is applied to prepare, for example, anti-biofouling membranes for water treatment facilities [36]. In the last case, enzymatic QQ prevents the formation of a stable bacterial biofilm on the membrane surface. The results of the current work are complying with this trend: according to SEM analysis, the building blocks of biofilm (i.e., cell aggregates) were efficiently disintegrated in the presence of NCs. The population of single cells is more susceptible to the action of antimicrobial agents and cannot attach to the surface with the same efficiency as aggregates. Such by-effect(s) may have the potential to be implemented in vitally important medical field(s), namely: to modify surfaces of implants, catheters, sutures, etc.

It is worth noting emodin (1,3,8-trihydroxy-6-methylanthracene-9,10-dione) is an anthraquinone derivative and is synthesized in multiple plants including those used for officinal and food purposes [30]. Particularly, the main sources for its production can be rhubarb (e.g., *Rheum rhabarbarum*), buckthorn (e.g., *Frangula alnus*), aloe (e.g., *Aloe vera*), etc., naringenin (5,7-dihydroxy-2-(4-hydroxyphenyl)chroman-4-one) belonging to flavanones [31] can be relatively readily isolated from citruses, tomatoes, etc. Thereby, these compounds are highly interesting from the standpoint of green chemistry, ecological compatibility and manufacturability/accessibility of sources.

Thus, the NCs of a well-combined QS effector and QQ enzyme modulates the effect of an antimicrobial peptide during combined application. Such complex antimicrobial composition has shown activity against both Gram-positive and Gram-negative bacterial strains. At the same time, bacterial cellulose used to study the antibacterial properties of NCs could be considered a useful prototype to implement a similar approach to other fibrous materials, which can be both dressing [20] and protective [26,37].

## 4. Materials and Methods

### 4.1. Materials

Bacterial cellulose (BC) was obtained from fructose-containing medium using immobilized *Komagataeibacter xylinum* B-12429 (All-Russian Collection of Microorganisms, Moscow, Russia) cells as described previously [37]. After separation from the medium, it was washed with 1 M NaOH and water, and then dried at room temperature overnight under sterile conditions. Before experiments, BC was cut into square pieces of 1 × 1 cm.

His_6_-OPH was expressed in recombinant *Escherichia coli* strain SG13009[pREP4] (Qiagen, Hilden, Germany) as described previously [37]. Further, it was isolated and purified with Ni-NTA agarose (Sigma-Aldrich, Darmstadt, Germany) according to the procedure [38]. Enzyme concentration was determined by Bradford assay with Coomassie Brilliant Blue G-250 (Sigma-Aldrich) and its purity was confirmed by sodium dodecyl sulfate polyacrylamide gel electrophoresis in a 12% polyacrylamide gel using a Mini-PROTEAN II cell (Bio-Rad, Hercules, CA, USA) followed by Coomassie Brilliant Blue R-250 (Sigma-Aldrich) staining. Enzyme activity was measured in a 0.1 M carbonate buffer (pH 10.5) as described previously [38] with an Agilent UV-8453 spectroscopy system (Agilent Technology, Waldbronn, Germany) at 405 nm using 8 mM paraoxon (Sigma-Aldrich) as a substrate. The initial linear parts of kinetic curves were used to calculate enzymatic activity. One unit of enzyme activity (OPH activity) was defined as the quantity of the enzyme necessary to hydrolyze 1 µmol of paraoxon per min at 25 °C.

### 4.2. Preparation and Analysis of Enzyme Nanocomplexes

To obtain enzyme NCs with polypeptides, a previously published method [24,27] was used. Briefly, 0.1 mg/mL of His_6_-OPH or penicillin acylase (Sigma-Aldrich) in PBS buffer (pH 7.4) was gently mixed with a water solution of PEG-PLE_50_ (Alamanda Polymers, Huntsville, AL, USA), PLE_50_ (Alamanda Polymers) or Gelofusine^®^ (B.Braun Medical AG, Sempach, Switzerland) in an equimolar ratio and filtered through a 0.2-μm Chromafil^®^ Xtra PES filter (Macherey-Nagel GmbH, Düren, Germany); 10 μg/mL naringenin or 1 μg/mL emodin (Sigma-Aldrich) in EtOH were added to the nanocomplexes at dosage 1 vol.% before filtering.

The hydrodynamic size of the NCs was determined as described previously [39] by PTA using a NanoSight NS500 instrument (Malvern Panalytical, Malvern, UK) equipped with an 80 mW 532 nm laser. The size distribution of nanoparticles was calculated using NanoSight software (ver. 2.3, Malvern Panalytical) on the basis of five independent experiments. Prior to analysis, enzyme NCs were diluted 10–100 times in filtered PBS buffer and the buffer alone was investigated under the same conditions as a negative control.

Silicon wafers for AFM were functionalized by 3-aminopropyltrimethoxysilane (APTMS, Sigma-Aldrich) according to the known protocol [25] with minor modifications. Briefly, 5 × 5 mm samples were sequentially and intensively washed with Pr^i^OH, piranha solution (H_2_SO_4_:H_2_O_2_ = 2:1) and water. After drying at 120 °C for 1 h, samples were immersed into a 0.25 vol.% APTMS in hexane (0.5 mL per 2 replicas) and gently shaken at 30 °C for 30 min. Then, the liquid phase was rapidly removed and 0.5 mL of CHCl_3_ was added and further shaken at 30 °C for 10 min. After that, the liquid was discarded and samples were washed two times with two portions of 2 mL water, with intermediate shaking at 30 °C for 30 min. Samples were dried at 90 °C for 30 min and stored at room temperature in closed vessels. A 5 μL sample of as-prepared enzyme NCs was applied per wafer in duplicate and exposed at room temperature for 5 min. After that samples were washed two times in 2 mL water, spin-dried and stored under vacuum before analysis.

The tapping mode of SmartSPM-1000 (AIST-NT, Novato, CA, USA) was used for obtaining the topography profiles. The diamond single crystal cantilevers AFM Probe ART™ D300 (SCDprobes, Tallinn, Estonia) used in this study had a typical spring constant of 40 N/m and a rounded tip of 5–10 nm radius. The spring constant of each cantilever was determined using a technique based on measuring the change in the resonant frequency of the fundamental mode of vibration [40]. Image analysis of grains distribution was performed in Gwyddion software (ver. 2.62, available at http://gwyddion.net/, accessed on 15 December 2022) [41]. A 50% threshold of maximal intensity (i.e., height) was applied and grains having an area less than 10 pixels (i.e., 10 nm^2^) were discarded also. Finally, the data were exported to OriginPro (ver. 9.4.2.380, OriginLab, Northampton, MA, USA) and envelopes were approximated under the assumption of Gaussian distribution (with or without asymmetrical modification).

### 4.3. Analysis of Antibacterial Activity

A 10 μL sample of 10 μg/mL naringenin or 1 μg/mL emodin (Sigma-Aldrich) in EtOH diluted 1–1000 times was loaded per BC sample (1 × 1 cm) and dried for 72 h at +8 °C under sterile conditions. The antibacterial activity was determined by the previously published procedure [20,42] with minor modifications using Gram-positive bacterial cells of *Bacillus subtilis* B-522 (All-Russian Collection of Microorganisms, Moscow, Russia) and Gram-negative bacterial cells of *Escherichia coli* DH5α (Thermo Fisher Scientific, Waltham, MA, USA). Briefly, 20 µL of (3 ± 1) × 10^6^ cells/mL in a 0.9% NaCl was loaded onto BC samples and exposed for 24 h at 25 °C. Then, samples were immersed in 1 mL of DMSO and gently stirred for 1 h. The residual ATP concentration in DMSO extract was determined using a standard luciferin–luciferase ATP reagent (Lyumtek Ltd., Moscow, Russia) according to the method [43] using a Microluminometer 3560 (New Horizons Diagnostic, Arbutus, MD, USA). The experiments were performed in triplicate. The calibration curves for transforming values of ATP concentration to colony-forming units (CFU) are presented in Figure S11.

A 10 μL sample of 10 mg/mL colistin or polymyxin B was applied per BC sample (1 × 1 cm) and dried for 24 h at 8 °C under sterile conditions. Then, 10 μL of 1 μg/mL naringenin or 0.1 μg/mL emodin in EtOH was loaded per the same samples and dried for 72 h at 8 °C under sterile conditions. A further 4 μL of 3 mg/mL penicillin acylase in PBS buffer (pH 7.4), or 5 μL of 0.15 mg/mL His_6_-OPH in PBS buffer, or a mixture of 4 μL penicillin acylase with 1.5 μL His_6_-OPH was applied to the same samples and dried for 24 h at 8 °C under sterile conditions. Thus, while omitting antibiotics, QS effectors, or enzymes, various compositions were prepared. All samples were simultaneously investigated towards *B. subtilis* and *E. coli* cells as described above.

To prepare samples for SEM analysis, a mature culture of *E.coli* cells was used. A 5 μL sample of 2.4 mg/mL penicillin acylase and 5 μL of 0.5 mg/mL His_6_-OPH in PBS buffer were mixed with 5 μL of 0.2 μg/mL emodin in H_2_O. Then, 10 μL of PBS buffer or 10 mg/mL polymyxin B was added. These mixtures were introduced to a 25 μL suspension of (3 ± 1) × 10^5^ cells/mL in a 0.9% NaCl. As a negative control, 25 μL of PBS buffer was used. All samples were gently shaken for 3 h at 25 °C. After that, a 5 μL of the sample was applied on an APS-modified silicon wafer and dried in the air at room temperature (ca. 30 min). Before SEM analysis, samples were freeze-dried (Freeze Dry System, Labconco, Kansas City, MO, USA), sputtered with gold and studied at various magnifications with a Supra 40-30-87 microscope (Carl Zeiss, Oberkochen, Germany).

### 4.4. Computational Methods

Crystallographic structures of acylase PvdQ from *Pseudomonas aeruginosa* (PDB 4M1J), carboxypeptidase A from *Bos taurus* (PDB 1YME) and protease (stearolysin or thermolysin) from *Geobacillus stearothermophilus* (PDB 6GHX) were obtained from the Protein Data Bank. The structure of His_6_-OPH was prepared previously [27] and based on PDB 1QW7.

Previously published procedures [21,27] were used to calculate the enzyme–ligand complexes. Briefly, the surface charge distribution of enzymes was calculated at pH 7.5 using the Adaptive Poisson–Boltzmann Solver (APBS) and PDB2PQR servers (ver. 1.4.2.1 and 2.1.1, respectively, available at http://www.poissonboltzmann.org/, accessed on 15 December 2022) with a PARSE force field and default settings [44]. Then, the structure was converted from the PQR to PDBQT format using AutoDockTools (as part of MGLTools ver. 1.5.6, available at https://ccsb.scripps.edu/mgltools/, accessed on 15 December 2022) [45].

Structures of QS effectors were drawn using ChemBioDraw software (ver. 12.0, CambridgeSoft, Cambridgeshire, UK) and then minimized using ChemBio3D with force field MM2. Further, the structures in the PDB format were converted to the PDBQT format using AutoDockTools with atomic charges calculated with the Gasteiger–Marsili method.

Enzyme–ligand complexes were simulated using AutoDock Vina (ver. 1.1.2, available at http://vina.scripps.edu/, accessed on 15 December 2022) [46] on a desktop computer equipped with an Intel Pentium Dual-Core CPU E5400@2.7GHz and 3 GB of available memory. The grid box was approximately centered on the center of mass of the enzyme. The size of the grid box was chosen so that any enzyme surface was within the box with an additional margin. Calculations were performed with default program options. Following the procedure, the ‘receptor’ (i.e., enzyme) was proposed as rigid and the ‘ligand’ (i.e., QS effector) was fully flexible. The best 12 poses with minimal energy were selected. The solvent-accessible area occupied by effectors on the surface of enzymes was visualized and calculated using the “*get_area*” function of PyMOL Molecular Graphics System (ver. 1.7.6, Schrödinger Inc., New York, NY, USA). Statistical analysis was performed using SigmaPlot (ver. 12.5, Systat Software Inc., San Jose, CA, USA), and the data are presented as means ± standard deviation (±SD) unless otherwise stated.

## 5. Conclusions

Thus, self-assembling NCs of QQ enzymes with QS effectors were designed, prepared and characterized in the work. Such NCs can be rationally preselected to minimize the negative influence on each other and were obtained via simple pooling of several components. The method can be promising to prepare enzyme NCs with different functionality.

## Figures and Tables

**Table 1:** Binding energy (affinity) of selected compounds to surfaces of enzymes.

Compound	Mean Binding Energy, kcal/mol				One-Way ANOVA
	His_6_-OPH	Penicillin Acylase	Carboxypeptidase A	Thermolysin	
Amentoflavone	−8.8 ± 0.6	−8.9 ± 0.3	−8.1 ± 0.7	−8.6 ± 0.7	*p* = 0.005
Apicidin	−6.4 ± 0.3	−6.8 ± 0.3	−6.1 ± 0.2	−7.4 ± 0.4	*p* < 0.001
DIPT	−4.2 ± 0.2	−4.3 ± 0.3	−4.1 ± 0.3	−4.3 ± 0.3	n.s. (*p* = 0.384)
Emodin	−7.5 ± 0.6	−7.2 ± 0.4	−6.5 ± 0.5	−6.9 ± 0.4	*p* < 0.001
Naringenin	−7.1 ± 0.5	−6.6 ± 0.2	−6.2 ± 0.3	−6.7 ± 0.4	*p* < 0.001
Ngercheumicin A	−7.0 ± 0.3	−7.1 ± 0.4	−5.3 ± 0.2	−6.4 ± 0.2	*p* < 0.001
Savirin	−8.5 ± 0.5	−7.9 ± 0.4	−7.8 ± 0.5	−8.0 ± 0.4	*p* = 0.001
UP5	−6.2 ± 0.2	−7.6 ± 0.2	−6.4 ± 0.5	−6.0 ± 0.3	*p* < 0.001
Amirinic acid	−7.3 ± 0.5	−7.0 ± 0.3	−6.4 ± 0.3	−7.4 ± 0.4	*p* < 0.001

**Table 2:** Calculated hydrodynamic sizes of main fractions of various enzyme NCs.

Enzyme	Polymer	QS Effector	Main Peak (nm)	Peak Range * (nm)
Penicillin acylase	PLE_50_	–	84 ± 20	42–127
		Emodin	177 ± 30	116–240
		Naringenin	152 ± 29	97–215
	Gelofusine	–	77 ± 15	47–108
His_6_-OPH	PLE_50_	–	85 ± 23	47–135
		Emodin	98 ± 23	57–147
		Naringenin	97 ± 20	58–138
	Gelofusine	–	80 ± 25	38–132

* It means that 90% of nanoparticles fell into this range under assumption of symmetric Gaussian distribution. Minor fractions were simulated also but were not presented in the metrics.

## Data Availability

The data presented in this study are available by request from the corresponding author.

## Supplementary Materials

The following supporting information can be downloaded at: https://www.mdpi.com/article/10.3390/ijms24031831/s1.
