# Supplementary material for: Self-Assembling Enzymatic Nanocomplexes with Polypeptides and Low-Weight Organic Compounds: Preparation, Characterization, and Application of New Antibacterials
Source: Int J Mol Sci. 2023 Jan 17;24(3):1831. doi: 10.3390/ijms24031831 (PMC9915939; doi:10.3390/ijms24031831)
Supplement: Supplementary file 1 [file ijms-24-01831-s001.zip › ijms-2141954-SI.pdf]

Supplementary materials

Self-assembling enzymatic nanocomplexes with polypeptides and low-weight organic compounds: preparation, characterization, and application of new antibacterials

Ilya Lyagin[1], Nikolay Stepanov[1], Denis Presnov[2], Artem Trifonov[3] and Elena Efremenko[1,*]

[1] Faculty of Chemistry, Lomonosov Moscow State University, 119991 Moscow, Russia
[2] Skobeltsyn Institute of Nuclear Physics, Lomonosov Moscow State University, 119991 Moscow, Russia
[3] Faculty of Physics, Lomonosov Moscow State University, 119991 Moscow, Russia

* Correspondence: elena_efremenko@list.ru; Tel.: +7-495-939-3170; Fax: +7-495-939-417

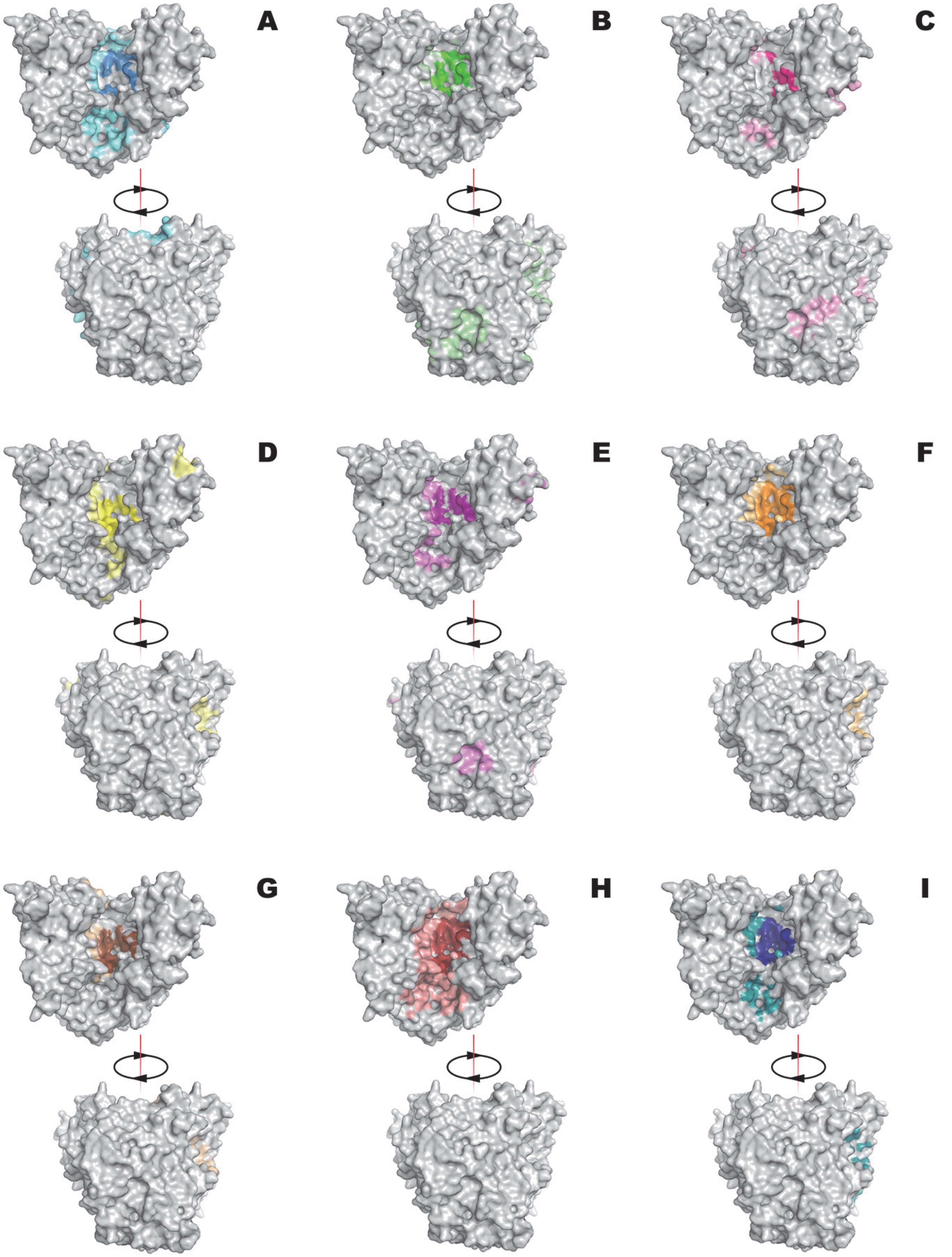

Figure S1. Binding of amentoflavone (**A**), apicidin (**B**), DIPT (**C**), emodin (**D**), naringenin (**E**), ngercheumicin A (**F**), savirin (**G**), UP5 (**H**) and amirinic acid (**I**) to penicillin acylase. Occupied surface near active site is highlighted by more intensive color. The top binding poses of emodin and naringenin near active sites are illustrated on Figure S4.

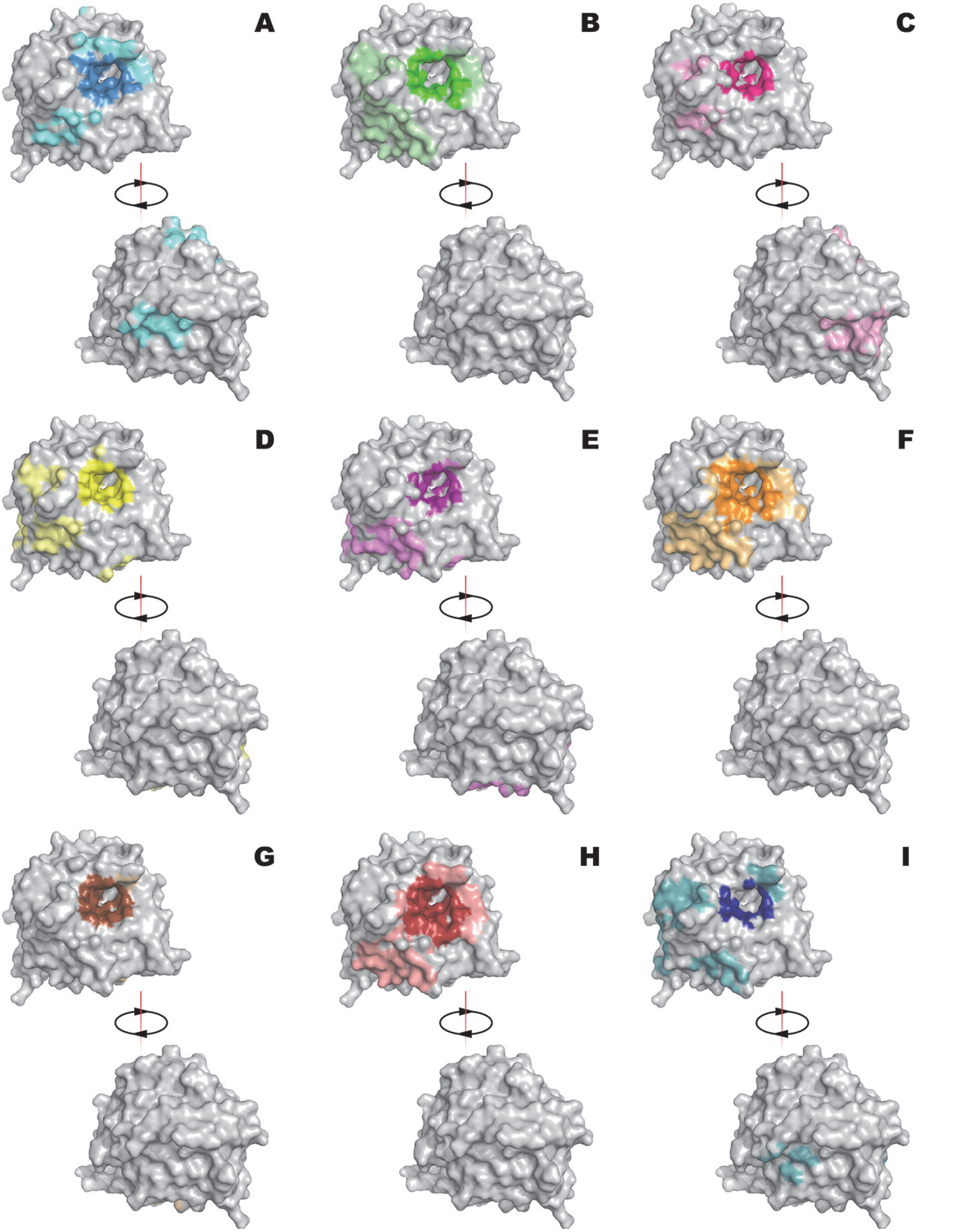

Figure S2. Binding of amentoflavone (**A**), apicidin (**B**), DIPT (**C**), emodin (**D**), naringenin (**E**), ngercheumicin A (**F**), savirin (**G**), UP5 (**H**) and amirinic acid (**I**) to carboxypeptidase A. Occupied surface near active site is highlighted by more intensive color.

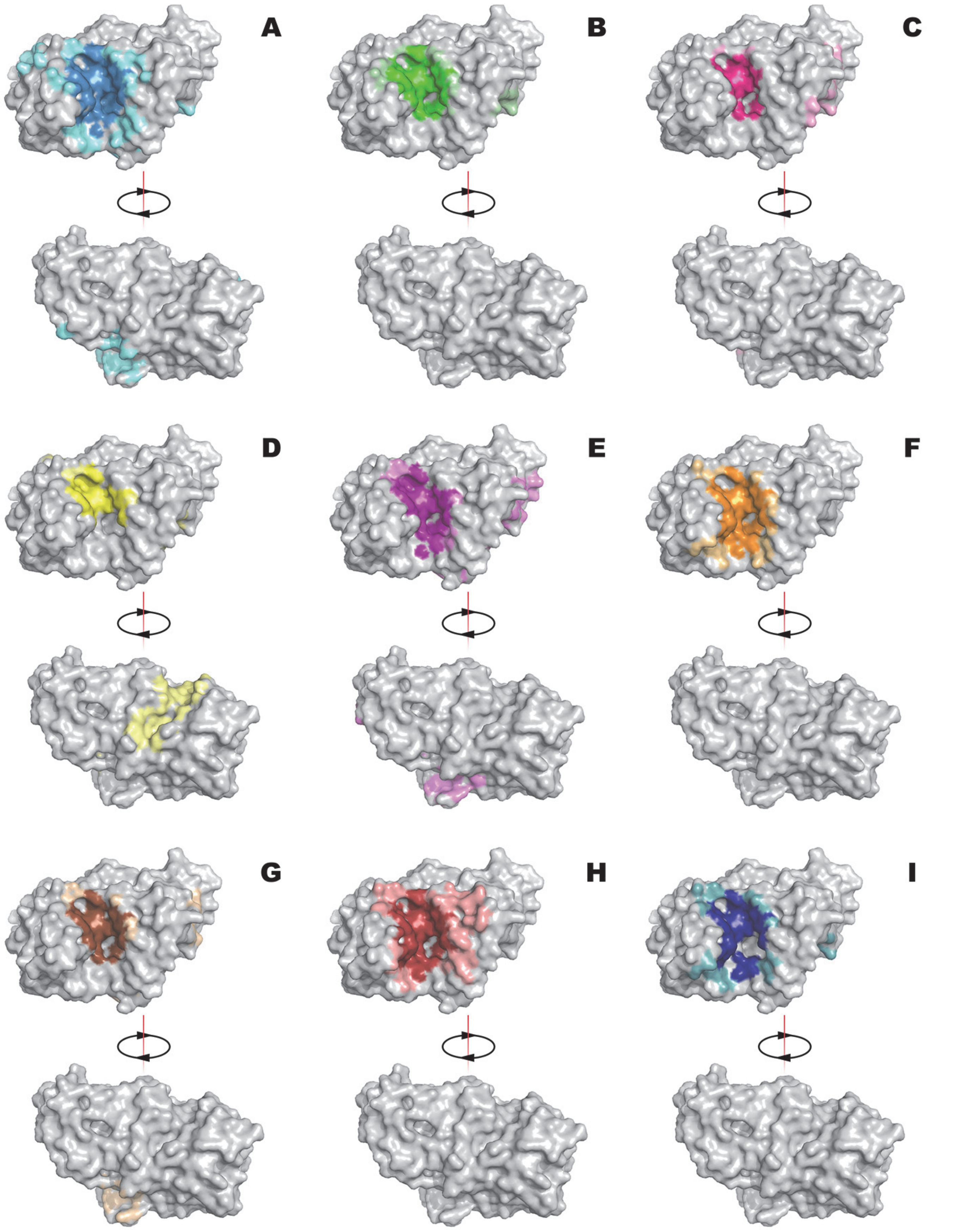

Figure S3. Binding of amentoflavone (**A**), apicidin (**B**), DIPT (**C**), emodin (**D**), naringenin (**E**), ngercheumicin A (**F**), savirin (**G**), UP5 (**H**) and amirinic acid (**I**) to thermolysin. Occupied surface near active site is highlighted by more intensive color.

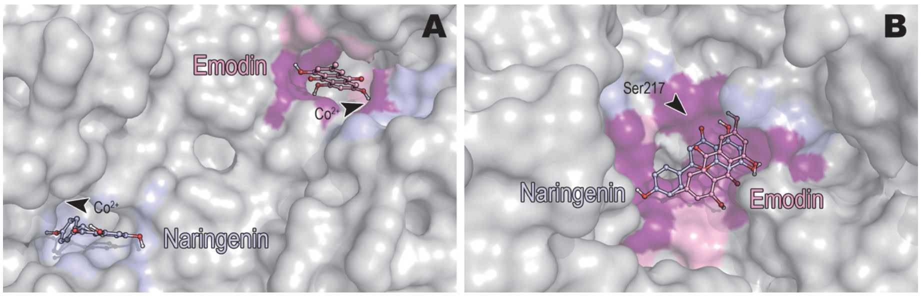

Figure S4. Enlarged view of top binding modes of emodin and naringenin affecting active sites of His6-OPH (**A**) and penicillin acylase (**B**). The surfaces occupied by certain compound near active sites are colored respectively while their intersection is colored violet. Catalytically essential atoms or group are marked by arrows.

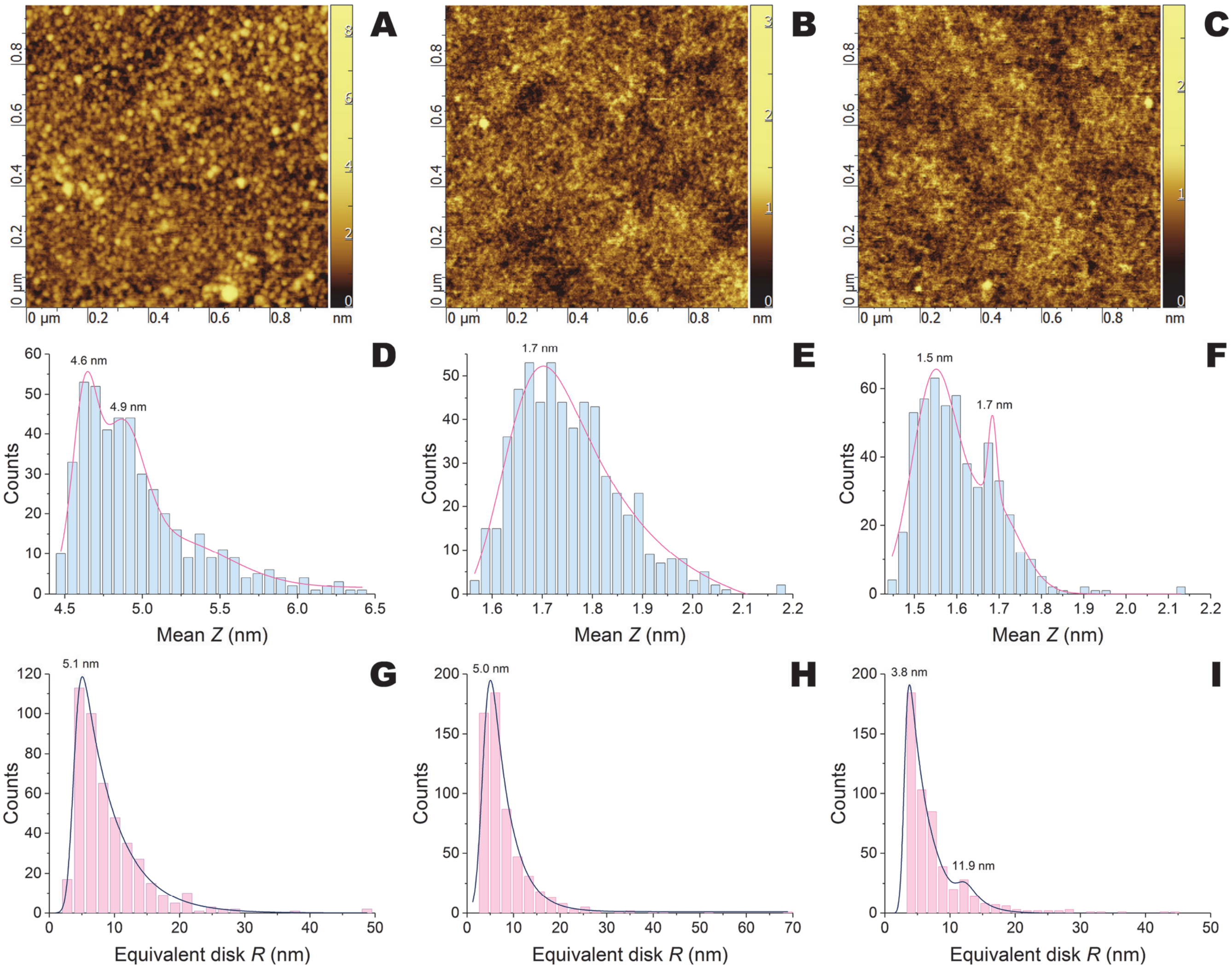

Figure S5. AFM images and their statistical treatment for silicon wafers before modification by APS (**A,D,E**), after modification by APS followed by usual washing (**B,E,H**) or prolonged washing (**C,F,I**). Mean height (Z) and equivalent disk radius (R) of grains were determined using Gwyddion (v. 2.62), while envelopes were approximated with OriginPro (v. 9.4.2.380) under assumption of Gaussian distribution (with or without asymmetrical modification). The most profound peaks are labeled.

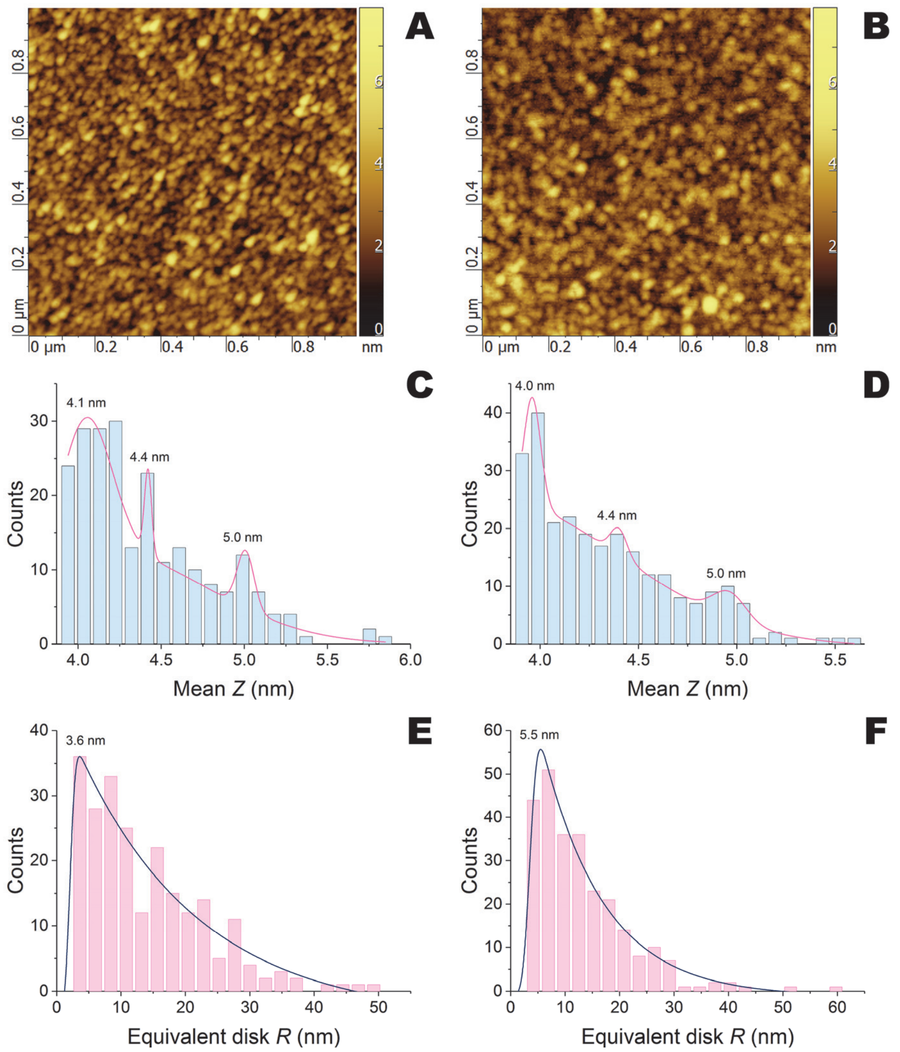

Figure S6. Reproducibility of AFM analysis and its statistical treatment. Silicon wafers were modified by APS and washed for 30 min and then His_6-OPH/PEG-PLE_{50} complexes were applied in two independent experiments before imaging in first (**A,C,E**) and second series (**B,D,F**).

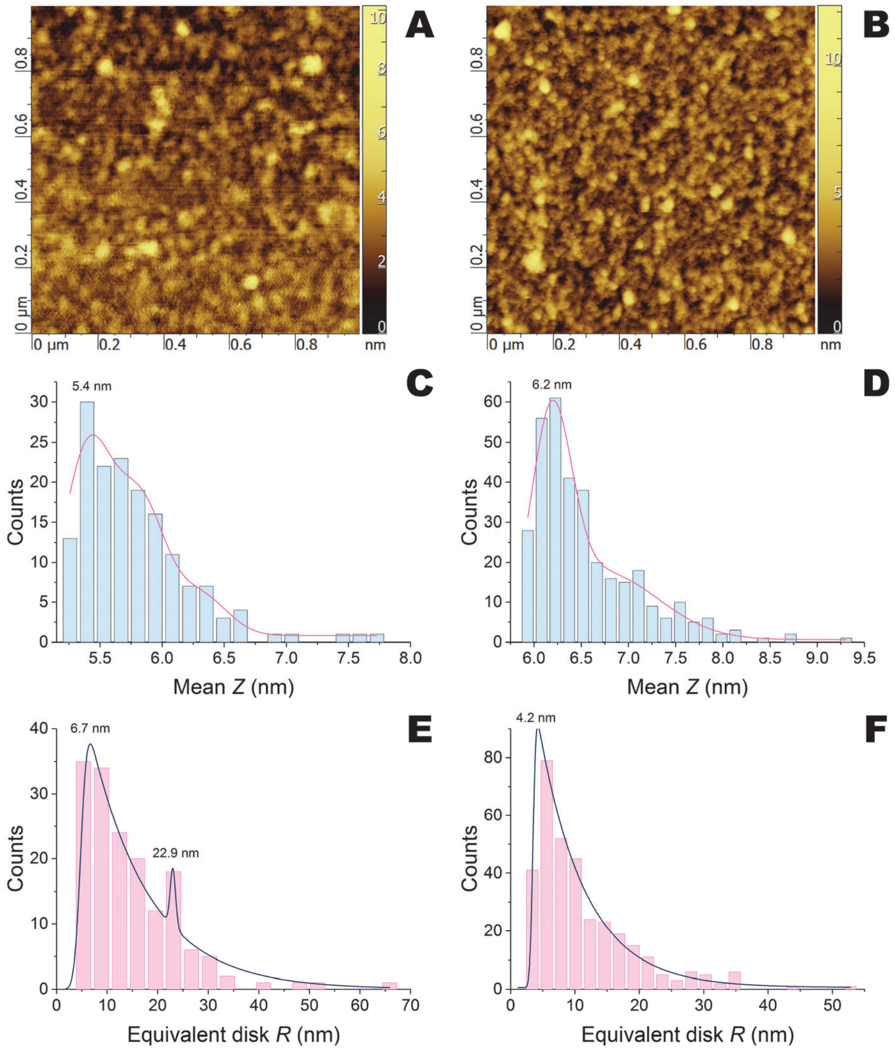

Figure S7. AFM images and their statistical treatment for APS-modified silicon wafers after application of penicillin acylase/gelofusine (**A,C,E**) and His_6-OPH/gelofusine complexes (**B,D,F**).

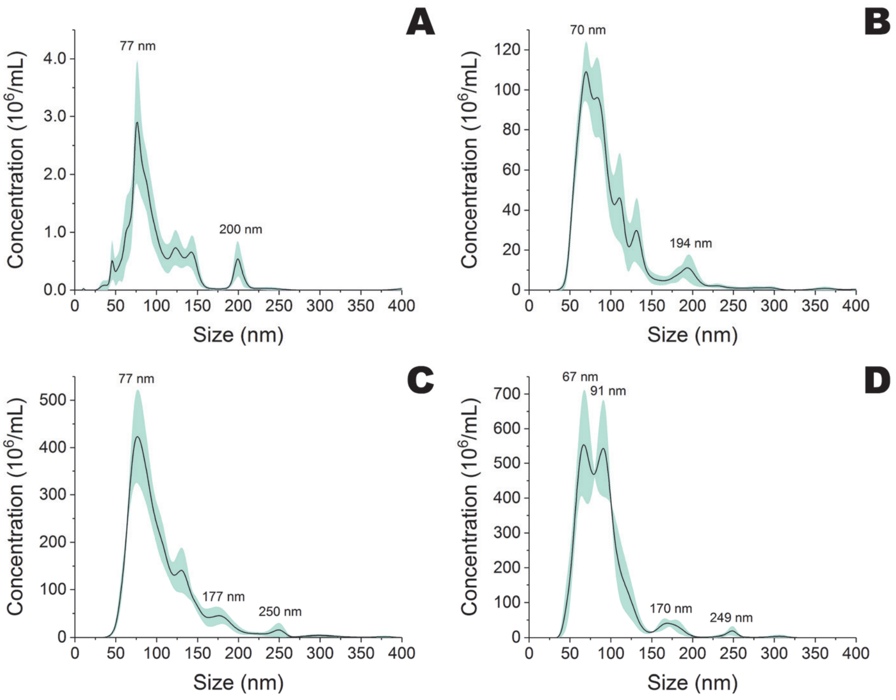

Figure S8. PTA of PBS buffer control (**A**), His_6-OPH/PEG-PLE_{50} (**B**), penicillin acylase/gelofusine (**C**) and His_6-OPH/gelofusine complexes (**D**). Mean values of 5 experimental runs are presented by line, while standard errors are filled by colorful areas. Some representative peaks are labeled.

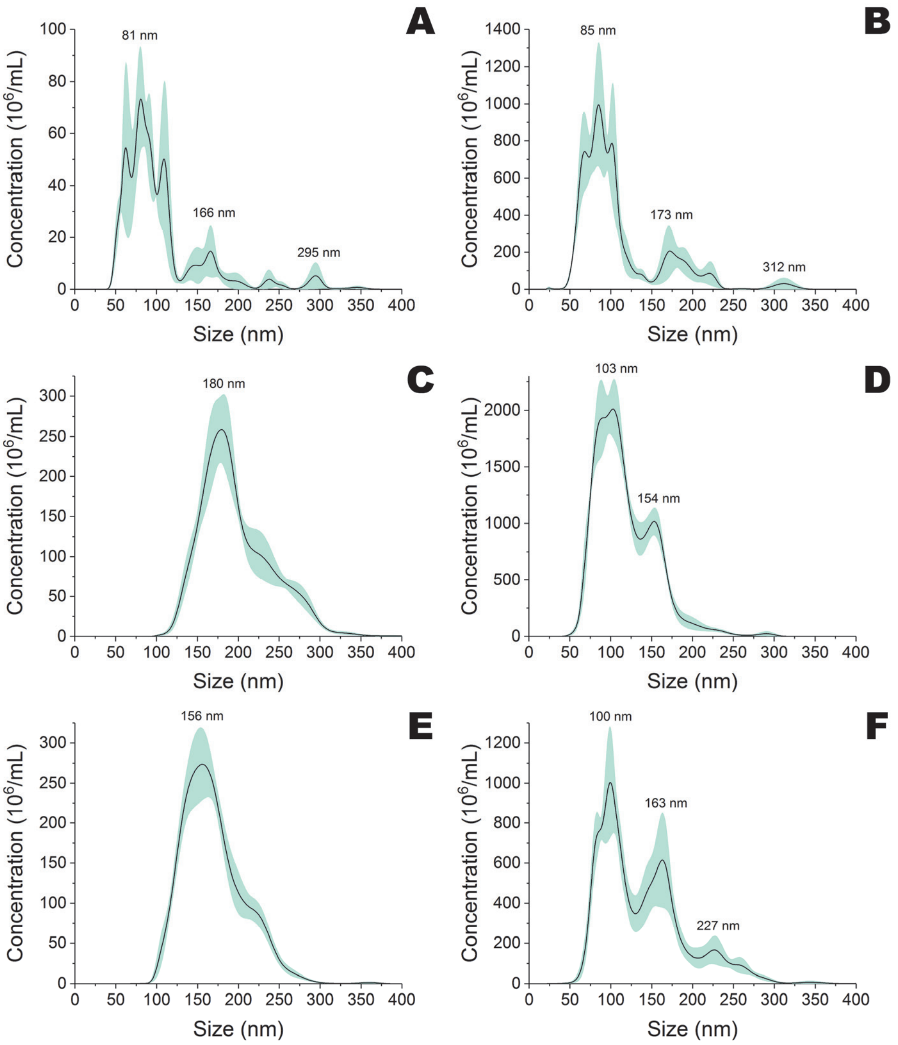

Figure S9. PTA of penicillin acylase/PLE_{50} (**A,C,E**) and His_6-OPH/PLE_{50} complexes (**B,D,F**) without QS effectors (**A,B**) and with emodin (**C,D**) or naringenin (**E,F**). Mean values of 5 experimental runs are presented by line, while standard errors are filled by colorful areas. Some representative peaks are labeled.

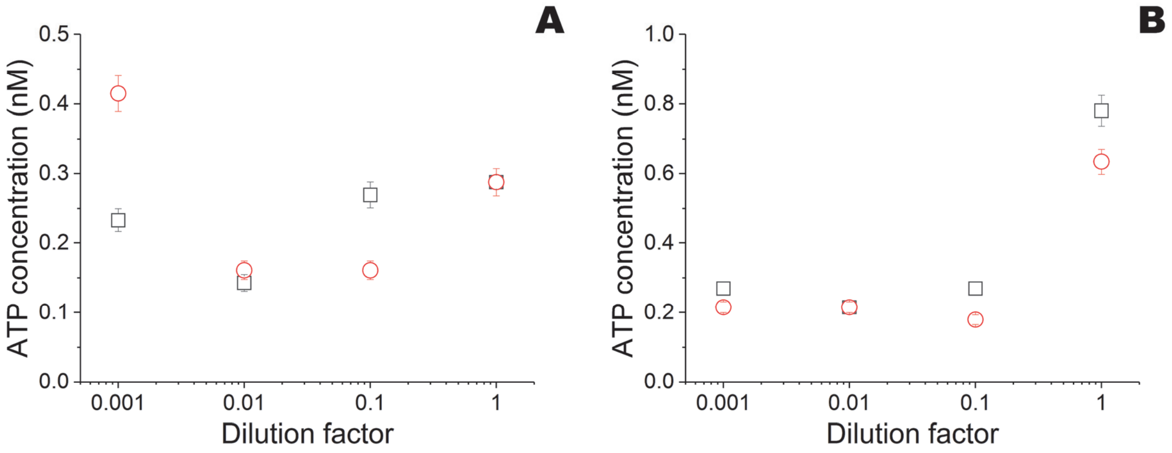

Figure S10. Antibacterial activity of emodin (□) and naringenin (○) towards *B.subtilis* (**A**) and *E.coli* (**B**) cells. An alcohol solution of 1 μg/mL emodin or 10 μg/mL naringenin diluted in 1–1000 times was applied to samples of bacterial cellulose (1 × 1 cm) followed by drying and measurement of its antibacterial activity according to known procedure [20].

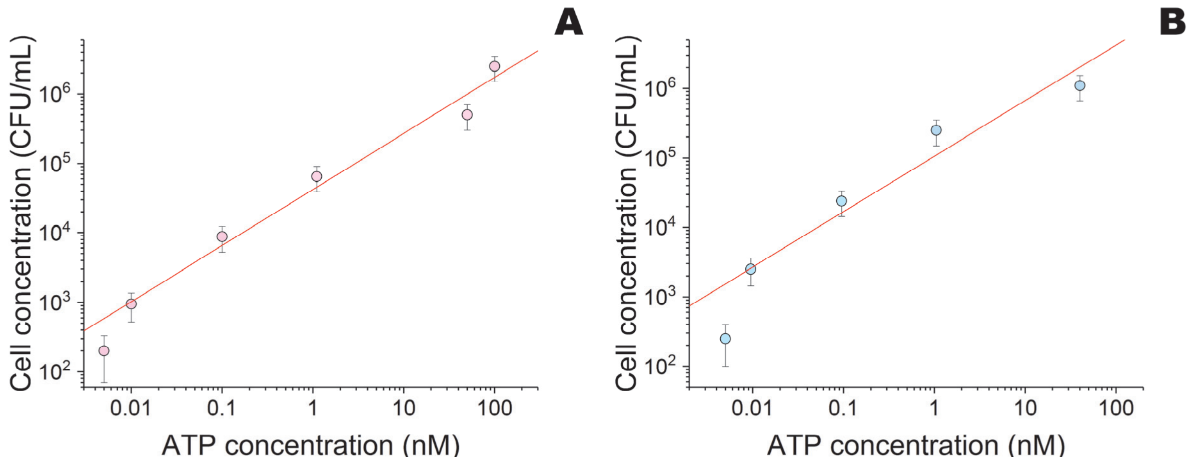

Figure S11. Calibration curves for transforming values of ATP concentration in *B.subtilis* (**A**) and *E.coli* (**B**) cells to colony forming units (CFU).

Table S1. Areas occupied by selected compounds on solvent-accessible enzyme surface.

Compound	Area occupied by compound near active site (or total), %				
	His_6-OPH	Penicillin acylase	Carboxypeptidase A	Thermolysin	median value
Amentoflavone	(8.7)	(11.3)	(14.9)	(19.1)	66.4 ± 20.6 (13.1 ± 4.5)
Apicidin	(9.6)	(12.3)	(12.7)	(8.9)	66.2 ± 16.5 (11.0 ± 1.9)
DIPT	(7.7)	(6.1)	(11.0)	(7.9)	35.3 ± 12.9 (7.8 ± 2.0)
Emodin	(5.6)	(7.8)	(13.3)	(9.3)	46.5 ± 22.4 (8.6 ± 3.2)
Naringenin	(7.7)	(6.0)	(13.0)	(11.7)	57.0 ± 20.2 (9.7 ± 3.3)
Ngercheumicin A	(9.9)	(6.6)	(13.6)	(8.7)	80.6 ± 27.4 (9.3 ± 2.9)
Savirin	(8.3)	(6.2)	(6.4)	(10.3)	61.0 ± 16.0 (7.4 ± 1.9)
UP5	(16.1)	(10.2)	(13.1)	(12.0)	82.7 ± 16.0 (12.6 ± 2.5)
Amirinic acid	(7.8)	(6.5)	(14.0)	(10.9)	57.9 ± 16.7 (9.4 ± 3.4)

Table S2. Multiple comparisons of different enzymes (data are from Table 1) by Holm-Sidak method.

Compound	Enzyme	vs. Enzyme		
		His_6-OPH	Penicillin acylase	Carboxypeptidase A
Amentoflavone	Penicillin acylase	n.s.		
	Carboxypeptidase A	$p = 0.017$	$p = 0.006$	
	Thermolysin	n.s.	n.s.	n.s.
Apicidin	Penicillin acylase	$p = 0.003$		
	Carboxypeptidase A	$p = 0.025$	$p < 0.001$	
	Thermolysin	$p < 0.001$	$p < 0.001$	$p < 0.001$
DIPT	Penicillin acylase	n.s.		
	Carboxypeptidase A	n.s.	n.s.	
	Thermolysin	n.s.	n.s.	n.s.
Emodin	Penicillin acylase	n.s.		
	Carboxypeptidase A	$p < 0.001$	$p = 0.007$	
	Thermolysin	$p = 0.031$	n.s.	n.s.
Naringenin	Penicillin acylase	$p = 0.023$		
	Carboxypeptidase A	$p < 0.001$	n.s. ($p = 0.050$)	
	Thermolysin	$p = 0.038$	n.s.	$p = 0.025$
Ngercheumicin A	Penicillin acylase	n.s.		
	Carboxypeptidase A	$p < 0.001$	$p < 0.001$	
	Thermolysin	$p < 0.001$	$p < 0.001$	$p < 0.001$
Savirin	Penicillin acylase	$p = 0.005$		
	Carboxypeptidase A	$p = 0.002$	n.s.	
	Thermolysin	n.s.	n.s.	n.s.
UP5	Penicillin acylase	$p < 0.001$		
	Carboxypeptidase A	n.s.	$p < 0.001$	
	Thermolysin	n.s.	$p < 0.001$	$p = 0.035$
Amirinic acid	Penicillin acylase	n.s.		
	Carboxypeptidase A	$p < 0.001$	$p = 0.002$	
	Thermolysin	n.s.	$p = 0.024$	$p < 0.001$

n.s. – non significant (i.e. $p > 0.05$)
